# Abnormal bile acid metabolism is an important feature of gut microbiota and fecal metabolites in patients with slow transit constipation

**DOI:** 10.3389/fcimb.2022.956528

**Published:** 2022-07-28

**Authors:** Yadong Fan, Chen Xu, Lulu Xie, Ying Wang, Shan Zhu, Jiren An, Yuwei Li, Zhikui Tian, Yiqi Yan, Shuang Yu, Haizhao Liu, Beitian Jia, Yiyang Wang, Li Wang, Long Yang, Yuhong Bian

**Affiliations:** ^1^ School of Integrative Medicine, Tianjin University of Traditional Chinese Medicine, Tianjin, China; ^2^ Department of Colorectal Surgery, Tianjin Union Medical Center, Tianjin, China; ^3^ School of Medicine, Nankai University, Tianjin, China; ^4^ The First Clinical College, Liaoning University of Traditional Chinese Medicine, Shenyang, China; ^5^ The Pharmacy Department, Tianjin Second People's Hospital, Tianjin, China; ^6^ Research Center for Infectious Diseases, Tianjin University of Traditional Chinese Medicine, Tianjin, China

**Keywords:** slow transit constipation, bile acid metabolism, 16S rRNA amplicon sequencing, metabolomics, diagnosis and intervention

## Abstract

Destructions in the intestinal ecosystem are implicated with changes in slow transit constipation (STC), which is a kind of intractable constipation characterized by colonic motility disorder. In order to deepen the understanding of the structure of the STC gut microbiota and the relationship between the gut microbiota and fecal metabolites, we first used 16S rRNA amplicon sequencing to evaluate the gut microbiota in 30 STC patients and 30 healthy subjects. The α-diversity of the STC group was changed to a certain degree, and the β-diversity was significantly different, which indicated that the composition of the gut microbiota of STC patients was inconsistent with healthy subjects. Among them, Bacteroides, Parabacteroides, Desulfovibrionaceae, and Ruminiclostridium were significantly upregulated, while Subdoligranulum was significantly downregulated. The metabolomics showed that different metabolites between the STC and the control group were involved in the process of bile acids and lipid metabolism, including taurocholate, taurochenodeoxycholate, taurine, deoxycholic acid, cyclohexylsulfamate, cholic acid, chenodeoxycholate, arachidonic acid, and 4-pyridoxic acid. We found that the colon histomorphology of STC patients was significantly disrupted, and TGR5 and FXR were significantly downregulated. The differences in metabolites were related to changes in the abundance of specific bacteria and patients’ intestinal dysfunction. Analysis of the fecal genomics and metabolomics enabled separation of the STC from controls based on random forest model prediction [STC *vs*. control (14 gut microbiota and metabolite biomarkers)—Sensitivity: 1, Specificity: 0.877]. This study provided a perspective for the diagnosis and intervention of STC related with abnormal bile acid metabolism.

## Introduction

Constipation is a common digestive system symptom with a reported prevalence of 7.0%–20.3% among Chinese adults (Peng et al., 2016; Long et al., 2017b). It is manifested by difficulty in defecation and/or reduced frequency of bowel movements, and dry and hard stools. With the accelerated pace of life, changes in dietary structure, and the influence of social and psychological factors, the prevalence of constipation is on the rise (Chu et al., 2014). It is more symptomatic and common in the elderly and women (Mugie et al., 2011; Vriesman et al., 2020). Slow transit constipation (STC) is the major category of intractable constipation and is characterized by colonic motility disorder (Hanson et al., 2019). The pathogenesis of STC mainly includes dietary structure with insufficient dietary fiber and water intake, lack of intestinal motility, abnormal enteric nervous system, dysfunction of colonic smooth muscle activity, and psychological factors (Rao et al., 2016; Vriesman et al., 2020). Treatments of functional constipation (three different subtypes: constipation with a normal transit, STC, and rectal evacuation disorders) include dietary interventions, educational and behavioral therapy, pharmacological interventions, transanal irrigation, neuromodulation, and imperative surgical interventions (Lacy et al., 2016; Vriesman et al., 2020).

New alternative therapies including fecal microbiota transplantation and acupuncture have been suggested to show the potential to treat functional constipation, but more supported studies are needed (Liu et al., 2016; Ding et al., 2018). The quality of life in patients with STC decreases, causing obvious economic and social burdens (Lacy et al., 2016; Vriesman et al., 2020). Destructions in the intestinal ecosystem are implicated with changes in STC, which suggest the possible roles of microbial disturbances in the development of constipation (Drossman, 2016; Parthasarathy et al., 2016; Ceresola et al., 2018). The structure of the intestinal microbiota is beneficial to the host metabolism, anti-inflammation, immunoregulation, and gastrointestinal motility (Nicholson et al., 2012; Kashyap et al., 2013; Propheter and Hooper, 2015; Si et al., 2018). This association can be explained by the regulation of the intestinal microbiota on the gastrointestinal motility, the osmotic effect of fermentation products, and metabolites, thus resulting in increased gas production (Hooper et al., 2001; Husebye et al., 2001). However, the role of gut microbes in the pathophysiology of STC is not fully understood.

Fecal genomics and metabolomics are useful tools for the quantification of the gut microbiome and related metabolites, which have more possible objective diagnostic potential. Thus, we performed 16S rRNA amplicon sequencing and ultra-high-performance liquid chromatography quadrupole time-of-flight mass spectrometry (UHPLC-Q-TOF-MS)-based metabolomics on fecal samples of patients with STC and healthy subjects to obtain evidence of distinct phenotypes of fecal microorganisms and metabolites. The relationship between different fecal microorganisms, metabolites, and clinical manifestations was explored according to the correlation analysis.

## Materials and methods

### Study subject recruitment

A total of 30 subjects met the Rome IV criteria for STC and 30 healthy controls were recruited from the outpatient department of Tianjin People’s Hospital from April 2020 through December 2020 (Drossman and Hasler, 2016; Rao et al., 2016). All subjects were evaluated by a physician. Inclusion criteria: STC patients confirmed by colonic transit stud with colonic transit time >48 h (Diamant et al., 1999); disease duration ≥ 3 months; age ≥ 18 years; body mass index (BMI): 18.5–25 kg/m^2^. Exclusion criteria: congenital megacolon; constipation caused by secondary intervention (e.g., drugs, metabolic disorder, endocrine disorders, or neurological disorders); previous abdominal surgery and perianal surgery; history or current of gastrointestinal diseases (e.g., malignancy and inflammatory bowel disease); infected with enteric pathogens; used prebiotics, probiotics, or proton pump inhibitors within the past month; pregnant or breast-feeding women; long-term smoking and/or alcohol addiction; confirmed to have hepatic, renal, cardiovascular, respiratory or psychiatric disease; suffered from other disease that could affect intestinal transit and the gut microbiota.

The researchers obtained metadata about each participant, including sex, age, gastrointestinal symptoms, dietary supplements, medications, and allergy history. All participants did not take antibiotics, probiotics, and prebiotics in the 3 months before the fecal sample collection. The quality of life of all subjects was evaluated according to the Wexner constipation score standard and the Gastrointestinal Quality of Life Index (GIQLI) (Eypasch et al., 1995; Agachan et al., 1996). All subjects or their families signed informed consents.

### Fecal sample collection and preparation

Standardized instructions and kits for collecting stool were provided to subjects, and the feces were collected and frozen in liquid nitrogen for cryopreservation immediately. The feces of the subjects were scored according to the Bristol stool form scale (BSFS) (Chumpitazi et al., 2016).

### DNA isolation and 16S rRNA amplicon sequencing

Total genome DNA from fecal samples was extracted using the CTAB/SDS method. DNA concentration and purity were monitored on 1% agarose gels. According to the concentration, DNA was diluted to 1 ng/μl using sterile water for further use. The V3–V4 variable region of 16S rRNA in fecal samples was amplified by PCR using a universal primer designed for the conserved region. According to the characteristics of the amplified 16S region, Illumina Miseq sequencing platform was used to construct a small fragment library and perform paired-end sequencing. Through the splicing and filtering of reads, cluster of operational taxonomic units (OTU) with Greengenes database (http://greengenes.lbl.gov/), species annotation, and abundance analysis, the species composition of the sample was revealed. Sequence analysis was performed by UPARSE software package using the UPARSE-OTU and UPARSE-OUTref algorithms. In-house Perl scripts were used to analyze α-diversity (within samples) and β-diversity (among samples). Sequences with ≥97% similarity were assigned to the same OTU. A representative sequence from each OTU was annotated taxonomic information using the RDP classifier (Wang et al., 2007).

### Analysis of species diversity, community structure, and differential microbes

We rarified the out table and drew four curves to evaluate the sequencing data including rarefaction curves, Shannon curves, rank-abundance curves, and species accumulation curves. The differences in α-diversity index between groups were analyzed by seven metrics: Observed Species, Shannon, Simpson, Chao 1, ACE, Coverage, and PD whole tree. Shannon index estimates flora diversity, and Chao1 estimates the species abundance. Principal component analysis (PCA) was applied to reduce the dimension of the original variables using QIIME (Bolyen et al., 2019). QIIME calculates both weighted and unweighted UniFrac distance, which are phylogenetic measures of β-diversity (Caporaso et al., 2010). The unweighted UniFrac distance was used for principal coordinate analysis (PCoA) to get principal coordinates and visualize them from complex, multidimensional data. At the same time, non-metric multi-dimensional scaling (NMDS) was also used to perform rank ordering and PCoA on the OTU data (Rivas et al., 2013). To confirm differences in the abundances of individual taxonomy between the two groups, STAMP software was utilized. Linear discriminant analysis effect size (LEfSe) was used for the quantitative analysis of differential microbes within STC and healthy subject groups (Segata et al., 2011). This method was designed to analyze data in which the number of species is much higher than the number of samples and to provide biological class explanations to establish statistical significance, biological consistency, and effect-size estimation of predicted biomarkers. Finally, PICRUSt software was used to predict the functional gene composition in samples, so as to analyze the functional differences between different samples or groups.

### Gut microbiome co-occurrence network analysis

In order to understand the correlations between different genera or species, a co-occurrence network was constructed based on the 16S rRNA data (Wang et al., 2018; Dan et al., 2020). The Spearman’s correlation coefficient was used to analyze the bacterial correlations in the STC and C groups according to the relative abundance of each species or genus, respectively. The significant correlations were visualized by Cytoscape version 3.7.1 (http://www.cytoscape.org) (Shannon et al., 2003).

### UPLC-Q-TOF-MS-based metabolomics

For fecal metabolomics, the fecal samples were slowly thawed at 4°C. An appropriate amount of samples was added to 800 μl of pre-cooled methanol/acetonitrile/water (2:2:1, v/v) solvent, vortexed to mix, sonicated at low temperature for 30 min, and stood at −20°C for 10 min. Subsequently, the samples were centrifuged at 14,000 g for 20 min at 4°C. The supernatant was dried in a vacuum centrifuge. For UPLC-Q-TOF-MS analysis, 100 μl of acetonitrile/water (1:1, v/v) solvent was used to dissolve the dry substances. The supernatant was taken for analysis, after being vortexed and centrifuged at 14,000 g for 15 min at 4°C. The samples were separated by UHPLC with Agilent 1290 Infinity LC (Agilent Technologies, Santa Clara, CA, USA). The 6600 Triple-TOF mass spectrometer (AB Sciex) was used to collect the first- and second-order spectra of the samples. The metabolite structure identification, data preprocessing, experimental data quality evaluation, and data analysis were performed in turn according to the peak area. Specific chromatography–mass spectrometry analysis conditions were carried out in accordance with the experimental procedures of Shanghai Applied Protein Technology (Shanghai, China).

Based on the fold change (FC) analysis and *t*-test/non-parametric test analysis, the difference analysis of all metabolites detected in the negative ion mode was performed.

After normalizing to total peak intensity, the processed data were analyzed by the R package, where it was subjected to orthogonal partial least-squares discriminant analysis (OPLS-DA) (Worley and Powers, 2013). The sevenfold cross-validation and response permutation testing were used to evaluate the robustness of the model. The variable importance in the projection (VIP) value of each variable in the OPLS-DA model was calculated to indicate its contribution to the classification. Metabolites with the VIP value >1 were further applied to Student’s *t*-test at univariate level to measure the significance of each metabolite; *p*-value less than 0.05 was considered as statistically significant. Kyoto Encyclopedia of Genes and Genomes (KEGG) pathway analysis was performed on the screened differential metabolites with VIP (VIP value ≥ 1 and *p*-value < 0.05).

### Random forest model prediction of potential diagnostic biomarkers

The receiver operating characteristic (ROC) curve and area under the curve (AUC) index were applied to optimize and verify the identified biomarkers (including gut microbiota and/or metabolites) and determine whether these biomarkers have diagnostic significance for STC (Falony et al., 2016; Liu et al., 2020). Random forest was used to build the diagnostic prediction models using the MetaboAnalyst 5.0 (https://www.metaboanalyst.ca) (Pang et al., 2021). To determine the association between Wexner constipation score, GIQLI, BSFS score, potential diagnostic gut microbiota, and metabolites, a correlation analysis was constructed using Spearman’s correlations.

### HE staining, immunohistochemistry, quantitative real-time polymerase chain reaction, and Western blot of colon tissues

Tissue samples of the control group were obtained from normal para-cancer colon tissue (more than 3 cm from the tumor) from colon cancer patients undergoing total colon resection. The tissue samples were confirmed to be normal colon tissue by two pathologic examiners. Colon tissues of the STC group were derived from patients who had to undergo surgery. All subjects or their families signed informed consents. All sample collection was performed in the operating room of Tianjin People’s Hospital and the samples were immediately stored at −80°C.

Paraffin sections (5 μm thick) of colon tissues from STC patients or healthy subjects were prepared after immersion in 4% paraformaldehyde for 4 h and 70% ethanol. The brief steps of HE staining included dewaxing the sections, impregnating the cytoplasm with eosin, redyeing the nucleus with hematoxylin, and dehydrating and sealing the tablets. The first step of immunohistochemistry was dewaxing and hydration. Permeabilize tissue cells by incubating with 0.1% Triton X-100 in phosphate buffered saline for 15 min on ice. Antigens of Takeda G protein-coupled receptor (TGR5, Lot: ab72608, Abcam, England) and nuclear farnesoid X receptor (FXR, Lot: bs-12867R, Bioss, China) were unmasked by microwaving sections in 10 mmol/L citrate buffer, pH 6.0 for 15 min. After blocking nonspecific proteins and endogenous peroxidase, the primary antibodies (1:200) and fluorescein-conjugated secondary antibody (1:100) were respectively incubated in a dark humidity chamber. Observe the results using a microscope in a dark room. Six to eight different fields were randomly selected from each group, and the number of TGR5- or FXR-positive cells was analyzed and calculated by ImageJ software (National Institutes of Health, United States). The lysate was used to extract the RNA from the colon tissues of two groups [Lot: DP431, Tiangen Biochemical Technology (Beijing) Co., Ltd.], and the RNA purity and concentration were detected by a spectrophotometer. cDNA was synthesized from 1 mg of total RNA using a reverse transcriptase kit [Lot: KR106, Tiangen Biochemical Technology (Beijing) Co., Ltd.]. The relative gene expression levels of *TGR5* and *FXR* were calculated by the 2^-△△CT^ method using the qRT-PCR method with *GAPDH* as the internal reference. Primer sequences are outlined in **Figure 5B**. The extraction of protein of the colon tissues was performed according to the kit instructions, and the protein concentration was determined using BCA assay. The quantified protein was pretreated and separated by SDS-PAGE, and transferred to polyvinylidene difluoride membranes. The membranes were blocked with bull serum albumin for 2 h at room temperature and incubated with rabbit antibodies against TGR5, FXR, or GAPDH (Lot: GB12002, Servicebio, China)(1:2,000) overnight at 4°C. The membranes were washed and further incubated with HRP-conjugated anti-rabbit or anti-mouse secondary antibody (1:10,000 dilution) for 1 h. The protein bands were developed using enhanced chemiluminescence reagent, imaged with a gel imaging system (Tanon, China), and quantified by NIH ImageJ software.

### Data analysis

PCoA, Fisher’s exact tests, Spearman correlations, and Wilcoxon rank-sum test were used to identify differences and the relations within the clinical characteristics. For normally distributed data, Student’s *t*-tests were performed to evaluate the differences in taxonomic abundance, Wilcoxon rank-sum test was performed when data were not normally distributed. *p*-value < 0.05 was considered statistically significant. The Benjamini–Hochberg method was used to correct *p*-value as false discovery rate (FDR) (Green and Diggle, 2007).

## Results

### Clinical characteristics of STC patients and healthy subjects

A total of 30 subjects with a clinical diagnosis of STC (age, 53.2 ± 16.323; sex, male:female, 7:23) and 30 healthy individuals (age, 47.967 ± 13.803; sex, male:female 9:21) were recruited (**Table 1**). There were no statistical difference in age, gender, and BMI between the two groups.

**Table 1:** Study participant demographics and characteristics.

Characteristic	Healthy Subjects	STC Patients	*p*-value
Subjects (*n*)	30	30	–
Age (years)	47.967 ± 13.803	53.2 ± 16.323	0.185 (*t*-test)
Male/Female	9/21	7/23	0.388 (Chi-square test)
Body mass index (kg/m^2^)	21.643 ± 1.704	22.076 ± 1.672	0.325(*t*-test)
Disease duration (years)	–	7.325 ± 8.559	–
Bristol Stool Scale	4.233 ± 1.073	2.5 ± 1.306	<0.001(*t*-test)
Wexner	3.767 ± 2.223	18.333 ± 7.439	<0.001(*t*-test)
GIQLI	127.967 ± 17.789	107.767 ± 21.394	<0.001(*t*-test)

### Alterations of gut microbiota composition and function in STC patients based on the 16S rRNA data

**Figure 1:**
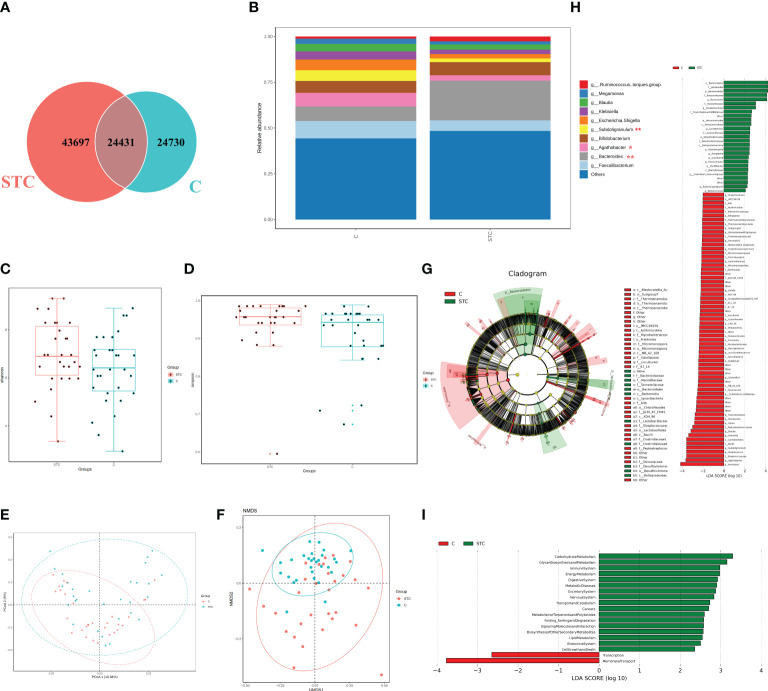
The shift of gut microbiome in slow transit constipation (STC) and control C subjects accroding to the 16S rRNA data. **(A)** Venn diagram of the observed OTUs in STC and C. **(B)** The relative abundance of the two groups at the genus level (Top 10). *p-value < 0.05 and ** *p*-value < 0.01, *t*-test. **(C, D)** Difference analysis of α-diversity index between the STC and C groups. Boxplot of difference between groups of Shannon index **(C)** with *p* = 0.094, *t*-test, and Simpson **(D)** with *p* = 0.021, Wilcoxon rank-sum test. **(E)** Principal coordinate analysis (PCoA) of the microbiota based on the unweighted UniFrac distance metrics for the STC and C groups. ANOSIM, *R* = 0.134, *p* = 0.001. **(F)** The differences between the STC and C groups were observed based on Non-Metric Multi-Dimensional Scaling (NMDS). **(G, H)** Cladograms generated by LEfSe indicating differences in the bacterial taxa between the STC and C groups. Red bars indicate taxa with enrichment in the C group, and green bars indicate taxa with enrichment in the STC group. **(I)** The LEfSe analysis of KEGG pathway (Welch’s *t*-test test).

The STC group and the healthy volunteer group displayed 43,697 and 24,730 unique OTUs, respectively. A total of 24,431 OTUs were shared by both groups (**Figure 1A**). The rarefaction curves, Shannon curves, rank-abundance curves, and species accumulation curves were generated from OTUs, with 97% identity achieved in all samples (**Figure S1**). This indicated that the testing samples were sufficient and the amount of data were reasonable for the investigation of fecal microbiota. Taking the genus level as an example, there was a difference in the relative abundance of species between the STC group and the healthy group. Bacteroides was significantly increased, while Agathobacter and Subdoligranulum were decreased in the STC group (*p*-value < 0.05 or 0.01) (**Figure 1B**). The results of 7 indexes in α-diversity analysis are shown in **Table 2** and **Figures 1C, D**. It is indicated that the α-diversity of gut microbiota in STC patients was richer than healthy subjects, but there were no significant differences except for the Simpson index (*p*-value = 0.021). The community composition structure and aggregation similarity of two groups were different based on the PCoA and NMDS of their microbiota (**Figures 1E, F**). The analysis results of LEfSe included cladograms (phylogenetic distribution) (**Figure 1G**) and histogram of LDA value distribution (LDA>2, **Figure 1H**). The LEfSe analysis results based on the prediction of KEGG pathway are shown in **Figure 1I**. The red and green nodes in the branches indicate the functional items and important microbial groups that play vital roles in the healthy or STC group. The differential microflora in the STC group was closely related to carbohydrate metabolism, glycan biosynthesis and metabolism, immune system, energy metabolism, digestive system, metabolic diseases, excretory system, nervous system, transport and catabolism, metabolism of terpenoid and polyketides, and lipid metabolism. There were significant differences in membrane transport (*p* = 0.037), cell motility (*p* = 0.047), and digestive system (*p* = 0.014) between two groups.

**Table 2:** α-diversity indices comparing STC patients to healthy subjects.

α-diversity index	Healthy Subjects	STC Patients	*p*-value
Shannon	6.351 ± 1.466	6.988 ± 1.425	0.094
Simpson	0.907 ± 0.082	0.942 ± 0.072	0.021
Ace	7,978.602 ± 3,979.623	9,247.546 ± 5,687.232	0.622
Goods coverage	0.974 ± 0.015	0.971 ± 0.0180	0.719
Chao1	7,766.749 ± 3,659.184	8,970.217 ± 5,284.232	0.602
Observed species	3,691.433 ± 1,705.291	4,347.967 ± 2,474.777	0.432
PD whole tree	99.107 ± 26.547	107.228 ± 36.495	0.676

Shannon index was tested by t-test, and the others were tested by Wilcoxon rank-sum test.

### Gut microbiota–based prediction of STC

**Figure 2:**
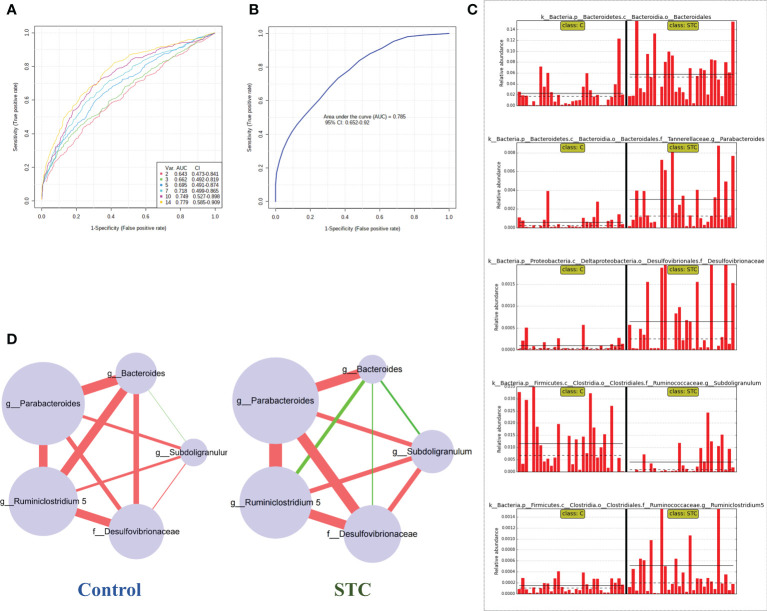
Random forest model prediction of potential diagnostic biomarkers of gut microbiota between STC patients and healthy subjects. **(A)** Classification performance of a random forest model using 16S rRNA abundance of 14 different bacterial genera or species. The cross-validation prediction performance of models with increasing number of predictors in order, and sorted by importance. **(B)** ROC curve displaying the classification for STC and C employing five potential diagnostic gut microbiota biomarkers (AUC = 0.785). **(C)** The abundance of 5 potential diagnostic gut microbiota biomarkers in each sample including g:Bacteroides, g:Parabacteroides, f:Desulfovibrionaceae, g:Ruminiclostridium 5, and g:Subdoligranulum. **(D)** Co-occurrence network of five potential diagnostic gut microbiota biomarkers in both the STC group and the control group based on the Spearman correlation algorithms. Each node presents a bacterial genus or species. The node size indicates the relative importance of each genus or species, and the density of the edges represents the Spearman coefficient. Red links stand for positive interactions between nodes, and green links stand for negative interactions.

To find and test the potential diagnostic gut microbiota biomarkers, a random forest model was applied based on the differential genus with relative abundance > 0 in at least 95% samples of two groups. With reference to the results of LEfSe analysis of different gut microbiota, we finally selected 14 different bacterial genera or families for performing the multivariate ROC curve based exploratory analysis under the automated important feature identification and performance evaluation (**Figure 2A**). After comprehensive consideration of the value of the AUC, five potential diagnostic gut microbiota biomarkers (g:Bacteroides, g:Parabacteroides, f:Desulfovibrionaceae, g:Ruminiclostridium 5, and g:Subdoligranulum) were screened out and were attempted to be constructed as a diagnostic model with AUC = 0.785 (**Figure 2B**). The relative abundances of these five bacterial genera in each group are shown in **Figure 2C**. In addition to g:Subdoligranulum, the other four diagnostic gut microbiota biomarkers were significantly upregulated in the STC group.

The human intestine tract has a complex microbial ecosystem, and the influence of individual groups in different microbial communities on the intestine tract is uneven (Yilmaz et al., 2019). In order to describe the potential relationship between the flora in the gut microbial community, we further constructed a co-occurrence network of five potential diagnostic gut microbiota biomarkers in the control and STC groups based on the significant Spearman correlations (**Figure 2D**). A different co-occurrence network was displayed between the five potential diagnostic markers. The variation trend relationship between them was visualized. There were 10 edges in total, whose density and colors represented the closeness of the relationship. The size of nodes represented its importance in the five bacterial communities.

### Metabolomics analysis revealed aberrant metabolic patterns in STC patients

**Figure 3:**
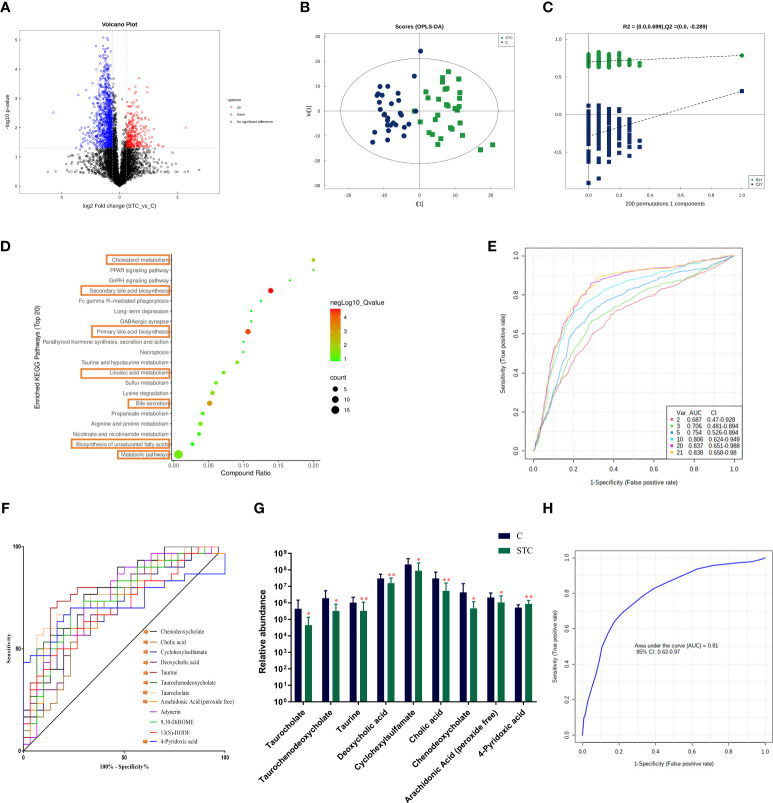
The fecal metabolites of STC patients and healthy subjects are significantly different. **(A)** The different metabolites were visualized in the form of volcano plot. The abscissa is the logarithm value of log2 of the fold change, and the ordinate is the logarithm value of −log10 of significance *p*-value (STC *vs*. C). Metabolites of difference with FC > 1.5, *p*-value < 0.05 are represented by rose red, while those with FC < 0.67 and *p*-value < 0.05 are shown in blue. Metabolites that are not significantly different are shown in black. **(B)** The score plot of orthogonal partial least-squares discriminant analysis (OPLS-DA), where t[1] represents principal component 1, t[2] represents principal component 2, and the ellipse represents the 95% confidence interval. The distribution of points reflects the degree of difference between groups and within group. The model evaluation parameter *Q* ^2^ obtained by sevenfold cross-validation is 0.311. **(C)** Permutation test of OPLS-DA. The *x*-coordinate represents the degree of permutation, and the *y*-coordinate represents the values of *R* ^2^ and *Q* ^2^. The green dot represents *R* ^2^, the blue dot denotes *Q* ^2^, and the two dashed lines represent the regression lines of *R* ^2^ and *Q* ^2^, respectively. **(D)** Top 20 enriched KEGG pathways of 21 screened differential metabolites (VIP value ≥ 1 and *p*-value < 0.05). **(E)** Classification performance of a random forest model using abundance of 21 differential metabolites based on multivariate ROC curve exploratory analysis. The cross-validation prediction performance of models with inreasing number of predictors in order, and sorted by importance. **(F)** The receiver operating characteristic curve (ROC) of 12 potential diagnostic biomarker metabolites [each area under curve (AUC) ≥ 0.7]. **(G)** The abundance of 9 different metabolites involved in the BA synthesis, metabolism, secretion, and lipid metabolism, which was believed to have diagnostic efficacy. Significance compared with the control group, **P < 0.01 or *P < 0.05 vs. the control group. **(H)** ROC curve displaying the classification for STC and C employing 9 diagnostic biomarker metabolites (AUC = 0.81).

Based on univariate analysis, differential analysis of all metabolites detected in negative ion mode was performed. The different metabolites with FC > 1.5 or FC < 0.67 and *p*-value < 0.05 were visually displayed in the form of a volcano plot (**Figure 3A**). There were more downregulated differential metabolites in the STC group than in the control group. The score plot of the OPLS-DA score showed that there was a large variation between the two groups (**Figure 3B**), and this evaluation model was stable with *Q* ^2^ = 0.311 (**Figure 3C**). VIP obtained from the OPLS-DA model could be used to measure the influent intensity and explanatory ability of the expression pattern of each metabolite on the classification and discrimination of each group of samples. Generally, metabolites with VIP value ≥ 1 and *p*-value < 0.05 were considered as differential metabolites to have a significant contribution to model interpretation. The information of the 21 differential metabolites selected is displayed in **Table 3**.

**Table 3:** The information of 21 significantly differential metabolites.

Name	VIP	Fold change	*p*-value	m/z	RT (s)	Quantitative analysis (Control)	Quantitative analysis (STC)
Thymine	2.004	1.976↑	0.023	125.03524	74.3435	484,330 ± 612,626	956,822 ± 924,101
Taurocholate	1.305	0.101	0.043	514.28179	197.259	439,651 ± 1,041,027	44,580 ± 88,504
Taurochenodeoxycholate	2.909	0.167	0.017	498.28652	145.579	1,912,584 ± 3,520,092	319,093 ± 512,153
Taurine	2.025	0.314	0.007	124.00744	291.676	1,029,081 ± 1,142,722	323,370 ± 770,728
Succinate	3.916	0.199	0.015	117.01962	391.109	3,013,974 ± 5,118,610	598,775 ± 1,387,217
Propionic acid	2.522	0.209	0.013	73.03003	391.176	1,221,545 ± 1,999,220	255,230 ± 550,046
Nervonic acid	1.253	1.878↑	0.027	365.34001	37.606	83,699 ± 61,566	157,186 ± 166,513
Glycyl-L-leucine	1.566	0.597	0.039	187.10837	283.697	687,000 ± 554,262	410,403 ± 456,281
Deoxycholic acid	12.233	0.525	0.008	391.28427	128.334	30,298,350 ± 23,615,390	15,918,241 ± 16,317,735
Cyclohexylsulfamate	26.333	0.417	0.039	178.0543	70.444	212,951,579 ± 270,482,964	88,817,956 ± 175,614,185
Cholic acid	14.581	0.259	0.009	407.27861	218.452	30,438,945 ± 42,352,355	7,880,015 ±16,816,427
Cholesteryl sulfate	1.793	1.643↑	0.009	931.61153	26.4205	285,135 ± 209,642	46,8399 ± 306,055
Chenodeoxycholate	5.894	0.107	0.045	783.57383	128.579	4,318,477 ± 10,292,191	461,818 ± 717,881
Arachidonic acid (peroxide free)	1.986	0.504	0.024	303.23131	38.7865	2,093,885 ± 1,879,405	1,055,170 ± 1,580,409
Adynerin	4.451	0.426	0.002	515.30189	52.427	4,542,108 ± 3,782,549	1,932,674 ± 2,039,784
9,10-DiHOME	2.347	0.436	0.002	313.23647	84.842	1,180,798 ± 1,006,075	514,772 ± 544,196
4-Pyridoxic acid	1.901	1.679↑	0.002	182.04518	44.08	514,197 ± 249,711	863,303 ± 529,983
2-Methylbenzoic acid	2.289	1.652↑	0.016	135.04488	129.02	635,109 ± 518,673	1,049,088 ± 747,583
1-Palmitoyl-2-hydroxy-sn-glycero-3-phosphoethanolamine	2.270	0.558	0.014	452.27705	194.122	4,105,361 ± 3,615,908	2,289,623 ± 1,488,993
1-Methylxanthine	1.211	0.583	0.028	165.04129	109.821	741,523 ± 618,759	432,025 ± 430,157
13(S)-HODE	1.308	0.534	0.015	295.2263	47.747	1,205,164 ± 1,081,968	643,204 ± 569,528

Mass-to-charge ratio (m/z); retention time (RT); quantitative analysis: response strength, means ± SD.

In order to clarify the functional changes related to specific differential metabolites in the stool of STC patients, we performed the KEGG pathway on the 21 screened metabolites (**Figure 3D**). The results showed that the KEGG pathway was mainly concentrated in secondary bile acid (BA) biosynthesis (−log_10_ *p*-value = 6.420), primary BA biosynthesis (−log_10_ *p*-value = 5.825), bile secretion (−log_10_ *p*-value = 4.266), cholesterol metabolism (−log_10_ *p*-value = 3.085), linoleic acid metabolism (−log_10_ *p*-value = 2.181), and biosynthesis of unsaturated fatty acids (−log_10_ *p*-value = 1.381).

The gut microbiota can modulate intestinal motility through the release of short-chain fatty acids (SCFAs) (Barbara et al., 2005; Martin-Gallausiaux et al., 2021). SCFAs produced by gut bacteria, especially butyrate and propionic acid, have multiple beneficial effects on host health, including the maintenance of mucosal integrity, the prevention of symbiotic expansion of potentially pathogenic bacteria in the gut and the regulation of energy metabolism through the gut–brain axis (Wang et al., 2019; Zhuang et al., 2019). The quantitative comparison analysis (response strength, means ± SD) of propionic acid and isobutyric acid in the feces of healthy subjects was 1,221,545 ± 1,999,220 and 1,563,167± 969,880, while in STC patients, they were reduced to 255,230 ± 550,046 and 1,814,435± 1,329,376 (*p*-value = 0.013 and 0.406). This indicated that the levels of SCFA changed to some extent, especially the decrease of propionic acid during the occurrence of STC, which was consistent with the previous results (Li et al., 2020; Tian et al., 2021).

We also compared the differences in the content of other secondary BAs or its conjugated BAs. In STC patients, except for lithocholic acid, all of them were reduced to varying degrees compared with healthy subjects, but the statistical differences were not significant (**Table 4**). Combined with differential metabolites, it was indeed found that primary and secondary BAs were significantly reduced in the feces of STC patients. Based on the above results and the roles of BAs in lipid metabolism (Fiorucci et al., 2021), we selected nine different metabolites that were deeply involved in BA synthesis, metabolism, secretion, and lipid metabolism, and demonstrated their abundance in the two groups (**Figure 3G**).

**Table 4:** The information of other secondary BAs or its conjugated Bas.

Name	VIP	Fold change	*p*-value	m/z	RT (s)	Quantitative analysis(Control)	Quantitative analysis (STC)
Glycolithocholic acid	0.196	0.752	0.380	432.30832	166.15	31,903 ± 38,586	23,986 ± 30,246
Lithocholic acid	13.188	1.106↑	0.713	375.28882	72.862	37,217,919 ± 40,433,719	41,149,879 ± 41,811,647
Taurolithocholic acid	0.185	0.727	0.334	482.29091	78.303	166,054 ± 203,462	120,664 ± 154,239
Glycochenodeoxycholate	0.405	0.319	0.017	472.30622	204.659	55,234 ± 83,234	17,642 ± 9,699
Glycodeoxycholic acid	1.079	0.119	0.139	450.31875	205.917	814,328 ± 2,616,266	96,808 ± 183,895
Tauroursodeoxycholic acid	0.338	0.665	0.193	482.29112	142.162	72,381 ± 92,688	48,152 ± 39,440
Taurochenodeoxycholate	0.519	0.427	0.053	500.30143	143.273	124,983 ± 194,069	53,315 ± 42,972

Mass-to-charge ratio (m/z); retention time (RT); quantitative analysis: response strength, means ± SD.

### Gut metabolite-based prediction of STC

To find the potential diagnostic metabolic biomarkers, the random-forest model was used to evaluate the accuracy of 21 differential metabolite abundances for the classification performance anmong STC patients and healthy subjects (**Figure 3E**). The AUC and 95% confidence intervals of the top 12 differential metabolites are shown in **Figure 3F**, which were regarded as potential diagnostic biomarker metabolites. Then, to create a biomarker model, the multivariate ROC curve-based exploratory analysis was performed. It is concluded that the prediction model containing nine differential metabolites [inculding taurocholate, taurochenodeoxycholate, taurine, deoxycholic acid, cyclohexylsulfamate, cholic acid, chenodeoxycholate, arachidonic acid (peroxide free), and 4-pyridoxic acid] deeply involved in BA synthesis, metabolism, secretion, and lipid metabolism showed a high discriminatory power to predict STC status with AUC = 0.81 (**Figure 3H**).

### Changes of morphology and bile acid-related receptor expression in colon tissues of STC patients

**Figure 4:**
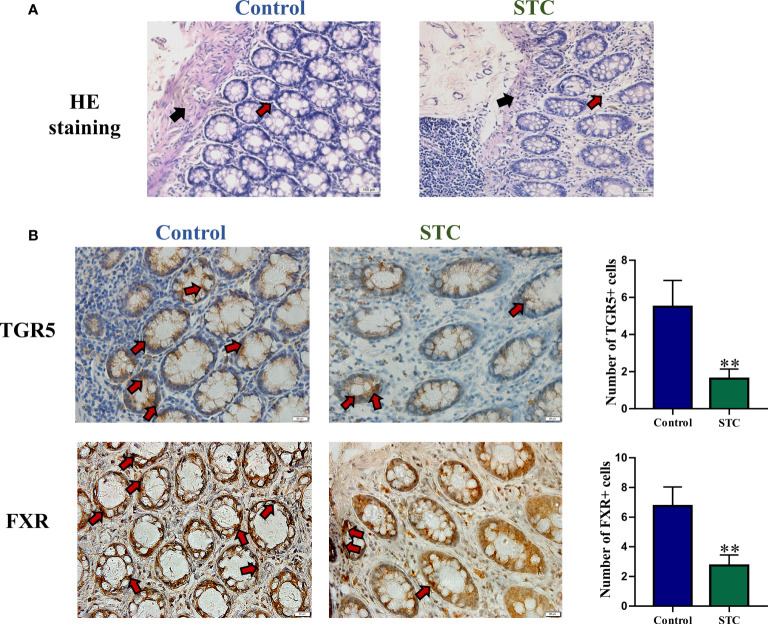
The morphology and BA-related receptor expression in colon tissues of STC patients and healthy subjects. **(A)** HE staining of colon tissues. Red arrows indicated inflammatory cell infiltration, and black arrows indicated mucosa layer structure of colon tissues. **(B)** Immunohistochemical staining results of TGR5 and FXR. The number of positive cells was quantitatively analyzed in six random selected fields of the same size using NIH ImageJ software. Significance compared with the control group, **p < 0.01 or *p < 0.05 *vs*. the control group.

**Figure 5:**
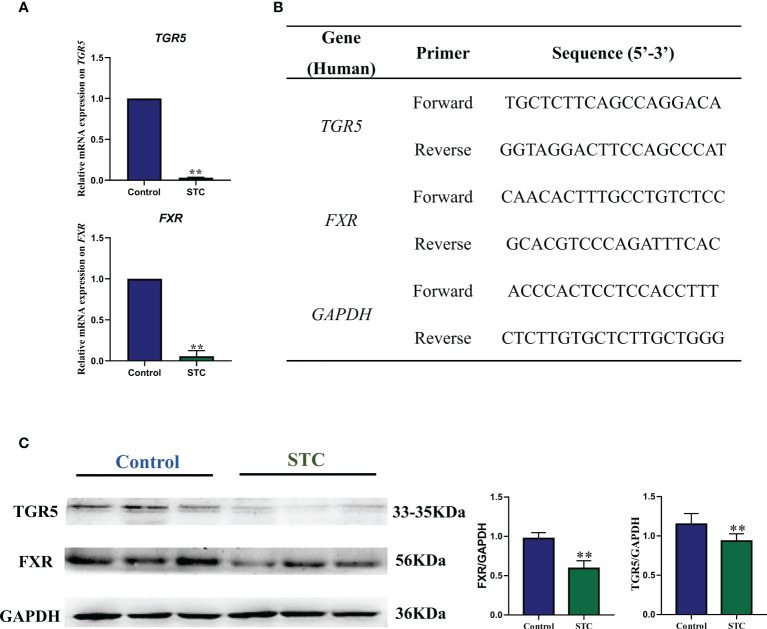
The gene and protein expression levels of TGR5 and FXR in colon tissues of STC patients and healthy subjects. **(A)** qRT-PCR results of gene expression of *TGR5* and *FXR*. **(B)** Gene primer sequence. The relative gene expression levels were calculated by the 2^-△△CT^ method with *GAPDH* as the internal reference (*n* = 6). **(C)** Western blotting results of protein expression of TGR5 and FXR. NIH ImageJ software was used to quantify the relative optical density of protein bands. Values are means ± SD (*n* = 6). The protein expression was detected in the same gel in which GAPDH was used as the internal control. Significance compared with the control group, **p < 0.01 or *p < 0.05 *vs*. the control group.

In healthy subjects, the epithelium of the colonic mucosa was intact. The glands were abundant and neatly arranged, and there was no obvious cytoplasmic expansion and inflammatory cell infiltration in the interstitium. The epithelium of the mucosal layer of STC patients was damaged or missing, with the number of glands reducing, whose cytoplasm was obviously enlarged. Compared with normal tissues, more inflammatory cells infiltrating were seen in the interstitium (**Figure 4A**).TGR5 is a cell membrane receptor located on human chromosome 2q35, and its homology is highly conserved in humans and mammals (>80%). TGR5 is highly expressed in immune cells, intestinal tract, and gallbladder (Duboc et al., 2014). FXR is a member of the nuclear receptor superfamily and a ligand-dependent transcription factor. It is mainly expressed in liver, intestine, kidney, and other tissues, which can regulate the metabolism and enterohepatic circulation of BAs (Fiorucci et al., 2010). The results of immunohistochemistry, qRT-PCR and WB experiments showed that compared with healthy controls, the gene and protein expression levels of TGR5 and FXR in the colon tissues of STC patients were significantly reduced (**Figures 4B** and **5**).

### Correlation analysis between Wexner constipation score, GIQLI, BSFS score, and diagnostic gut biomarkers

**Figure 6:**
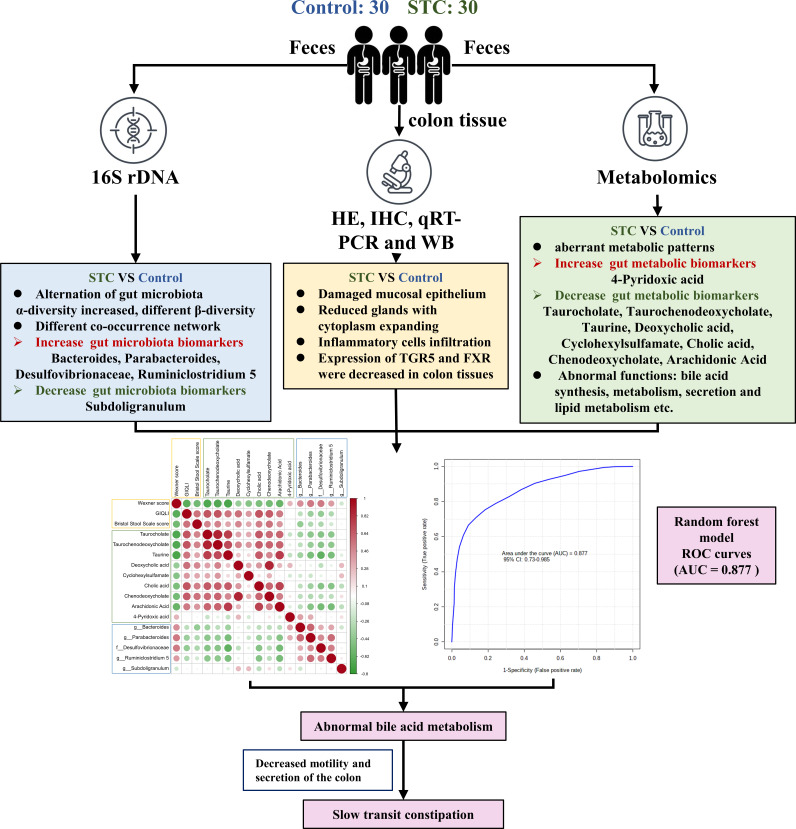
The summary of gut microbiota composition and metabolism analysis between STC and healthy subjects. Compared to the healthy subjects, the relative abundance of Bacteroides, Parabacteroides, Desulfovibrionaceae, and Ruminiclostridium 5 were increased while Subdoligranulum was decreased significantly in feces of STC patients. STC patients displayed alternation of gut microbiota composition. STC patients displayed decreased level in metabolites that were associated with BA synthesis, metabolism, secretion, and lipid metabolism. Experimental studies on human colon histology showed decreased expression of TGR5 and FXR in STC patients. Wexner constipation score was negatively correlated with these diagnostic metabolites, while GIQLI and BSFS scores were positively correlated. There was obvious positive correlation among metabolites. There was a certain negative correlation between the differential microbiota and the differential metabolites. Comprehensive use of 14 diagnostic gut microbiota and metabolite biomarkers may accurately distinguish STC and healthy population with AUC = 0.877.

Correlation analysis can help measure the closeness of significant diagnostic gut biomarkers (microbiota and metabolites) to clinical manifestations. The results are shown as heatmap in **Figure 6**. Red indicated positive correlation, green indicated negative correlation, and white indicated non-significant correlation. The color depth was related to the absolute value of the correlation coefficient. The significance of the correlation was related to the size of the point. The smaller the *p*-value was, the higher the significance was and the larger the point was. Compared with microbiota, Wexner constipation score, GIQLI, and BSFS score were more correlated with diagnostic gut metabolites. Wexner constipation score was negatively correlated with these diagnostic metabolites, while the other two were positively correlated. There was obvious positive correlation among metabolites. On the whole, there was a certain negative correlation between the differential microbiota and the differential metabolites, which provided enlightenment for us to study the regulatory relationship between the two. Comprehensive use of 14 diagnostic gut microbiota and metabolite biomarkers may accurately distinguish STC and healthy population with AUC = 0.877.

## Discussion

As shown in the summary of **Figure 6**, compared to the healthy subjects, the relative abundance of Bacteroides, Parabacteroides, Desulfovibrionaceae, and Ruminiclostridium 5 was increased while Subdoligranulum was decreased significantly in feces of STC patients. STC patients showed increased α-diversity.The β-diversity of the two groups was remarkably different, which displayed alternation of gut microbiota composition. Furthermore, STC patients displayed a decreased level in metabolites that were associated with BA synthesis, metabolism, secretion, and lipid metabolism. Experimental studies on human colon histology also confirmed this. The random forest prediction model was used to distinguish STC patients from healthy people by using few specific significant diagnostic gut microbiota and metabolites, which had a medium degree of reliability. It provided the possibility of generating a supplementary method for the risk assessment of the intestinal health monitoring model.

STC is a chronic disabling disease characterized by delayed colonic transit without outlet obstruction. It is refractory to drugs and finally treated with colectomy as the disease progresses (Knowles et al., 2017). Gastrointestinal motility is the key to the normal function of the human gastrointestinal tract. Over the years, many studies have been conducted on the mechanism of STC (Mazzone et al., 2020). Although the pathogenesis of STC has been partially elucidated, it is not completely clear. STC has complex pathogenesis, and most studies focus on enteric nerve and muscle diseases, abnormalities of neurotransmitters, interstitial cells of Cajal and enteric glial cell as well as chloride channel dysfunction. In recent years, the correlation between organism flora and diseases has gradually become an important means of disease mechanism research and a new perspective of disease treatment. Under the physiological conditions, the types and proportions of gut microbiota maintain a dynamic balance, which interact with the host to exert essential functions such as immunity, nutrition, immunity, and metabolism (Fiers et al., 2020; Schwabe and Greten, 2020). Studies have found significant differences in the composition of intestinal mucosa and fecal flora between STC patients and healthy controls (Ding et al., 2018; Zhang et al., 2018; Tian et al., 2021). Compared with normal subjects, the structure, abundance, and co-occurrence of gut microbiota were significantly altered in STC patients in our study, which was consistent with the characteristics of microbiota in children with chronic functional constipation (de Meij et al., 2016). The opportunistic pathogen Parabacteroides can induce depressive-like behavior in a mouse model of Crohn’s disease, whose relative level can be reduced by dietary synbiotic to ameliorate constipation (Gomez-Nguyen et al., 2021; Yang et al., 2021). As an important endotoxin producer in patients with constipation, depressed Desulfovibrionaceae bacteria was benificial to promoting intestinal hormone secretion and maintenance of intestinal barrier integrity (Zhuang et al., 2019). It is worth noting that studies have shown that Subdoligranulum is negatively correlated with clinical symptoms and inflammation of inflammatory bowel disease, and may be a potential probiotic for its treatment (Kim et al., 2021; Xia et al., 2021).

Metabolic pathways encoded by the human gut microbiota continuously communicate with the host through a large number of biologically active metabolites (Postler and Ghosh, 2017). BAs have both hydrophilicity and hydrophobicity, which can effectively reduce the surface tension between lipid and water phases to facilitate absorption of fat-soluble vitamins and lipids (Ridlon et al., 2006). Primary BAs are synthesized in hepatocytes using cholesterol as raw material under the catalysis of 17 biochemical enzymes, and excreted into the duodenum with bile. About 95% of BAs pass through the apical sodium-dependent bile acid transporter (ASBT) of intestinal cells in the terminal ileum, which compensate for the lack of BA synthesis ability of hepatocytes. The intestinal symbiotic bacteria convert some BAs (approximately 5%) entering the colon into various intestinal BAs, which are vital hormones regulating host energy balance and cholesterol metabolism through several G-protein-coupled receptors and/or nuclear receptors (Fiorucci and Distrutti, 2015; Wahlstrom et al., 2016). Primary BAs include cholic acid (CA) and chenodeoxycholic acid (CDCA) (Alrefai and Gill, 2007). In the colon, bacterial 7α-dehydroxylase (mainly derived from *Clostridium* spp. Eubacteria and *Clostridium* spp. XIVa) removes 7α-OH groups from CA and CDCA to form deoxycholic acid (DCA) and lithocholic acid (LCA), respectively. CA, CDCA, DCA, and LCA are collectively referred to as free BAs, which are combined with glycine and taurine to produce conjugated BAs (Alrefai and Gill, 2007; Vallim et al., 2013; Appleby and Walters, 2014). In this study, we found that the feces of patients with STC decreased significantly in primary and second BAs especially including cholic acid, chenodeoxycholate, taurine, taurocholate, taurochenodeoxycholate, deoxycholic acid, cyclohexylsulfamate, and glycochenodeoxycholate, indicating that the metabolism of BAs in the patient’s body was abnormal. BAs can also activate phospholipase 2 by interfering with the cell membrane, causing the cell to release arachidonic acid, promoting the production of reactive oxygen species, and inducing DNA damage (Sanchez, 2018). The reduction of arachidonic acid (peroxide free) is consistent with previous studies. Furthermore, the study of SCFAs as important metabolites in intestinal diseases has received widespread attention. Our study also found a significant reduction in propionic acid during STC, but significant changes in other types of SCFAs were not observed.

The action of BAs in the colon is known as a “physiological laxative”. When the reabsorption of BAs in the ileum is insufficient and hepatoenteric circulation is broken, more BAs will be transported to the colon (Jiang et al., 2015). BAs can activate intracellular adenylate cyclase, increase the permeability of the intestinal mucosa, promote intestinal electrolyte and water secretion, strengthen colonic transmission, and stimulate defecation (Chedid et al., 2018). Clinical studies have indicated that an increase in the total amount of fecal BAs in patients is significantly associated with accelerated colonic transit (Bajor et al., 2015; Dior et al., 2016). Animal studies have shown that BAs directly induce accelerated colonic motility (Traub et al., 2008; Kim et al., 2017). Therefore, supplementation of the proper amount of specific BA analogs or the use of drugs that inhibit the reabsorption of ileal BAs is beneficial to improve the clinical symptoms of patients with constipation, and has become one of the new treatment options for the treatment of STC (Jiang et al., 2015). Elobixibat is a highly selective ASBT inhibitor whose mechanism of action is to reduce the ileum reabsorption of BAs and increase the concentration of BAs entering the colon. Elobixibat resolved constipation in the short term (10 mg/day for 2 weeks) and was well tolerated with short-term and long-term (5 mg/day or 15 mg/day, or maintain the 10 mg/day dose for 1 year) treatments resulting from a randomized, double-blind, placebo-controlled, phase 3 trial and an open-label, single-arm, phase 3 trial (Nakajima et al., 2018). In a double-blind placebo randomized controlled study of oral sodium CDCA (500 mg/day or 1,000 mg/day for 4 days) in the treatment of 36 female patients with constipation-predominant irritable bowel syndrome, it was found that compared with the control group, treatment with sodium CDCA could improve the clinical symptoms including accelerating the transit of the entire colon, increasing the defecation of the patients, and softening the feces to make it easier to excrete from the body (Rao et al., 2010). Evidence supported the use of increasing the concentration of endogenous BAs to treat chronic constipation. However, there are no specific reports of this strategy on the therapeutic effect of STC.

The hepatic BAS synthesis rate is reversibly controlled by a feedback mechanism of FXR mediated in the liver and ileum. The rate of hepatic BA synthesis is reversibly controlled by an FXR-mediated feedback mechanism in the liver and ileum (Galman et al., 2003). The intestinal FXR activity maintained good outflow of BAs back to the portal vein and controled the reuptake of BAs into enterocytes (Stanimirov et al., 2015). After knocking out the *FXR* gene in the intestinal tract of wild mice, there was a significant inflammatory response in the intestinal tract of the mice, and the mRNA expression of inflammatory cytokines increased significantly (Vavassori et al., 2009). A large number of animal studies have confirmed that the activation of intestinal FXR can inhibit the transcriptional activity of NF-κB (Modica et al., 2008; Gadaleta et al., 2011; Miyazaki et al., 2021). The regulation of liver and intestine FXR activity reduced inflammation and epithelial permeability by lowering the levels of proinflammatory mediators in the gut. In addition, intestinal FXR activation induced transcription of genes involved in intestinal protection to prevent bacterial invasion and epithelial damage (Mosińska et al., 2018; Biagioli et al., 2021). Compared to FXR, TGR5 may be activated by hydrophobic BAs (taurine further enhances its potency) to promote permanent signaling transmission. The activation of TGR5 in colonic epithelial cells could suppress cholinergic-induced secretory responses and basal secretory tension (Cozma-Petruţ et al., 2017; Long et al., 2017a). Studies have shown that overactivation of epithelial TGR5 by lipophilic BAs, such as DCA and LCA, promoted colonic motility and led to diarrhea through a 5-HT-mediated pathway. In the mice lacking TGR-5, shortened transit time and increased constipation were reported (Alemi et al., 2013). These results suggested that changes in colonic TGR5 expression could alter small intestinal and colonic transit (Mosińska et al., 2018). Moreover, BA-dependent TGR5 activation significantly inhibited the activation of NF-κB in wild-type mice injected with lipopolysaccharide compared with TGR5-deficient mice (Wang et al., 2011). FXR and TGR5 agonists are promising in controlling inflammation in Crohn’s disease and ulcerative colitis (Yoneno et al., 2013; Perino and Schoonjans, 2015; Jia et al., 2018). In our study, we found that the expression of TGR5 and FXR in colon tissues was significantly downregulated. On the one hand, it reflected the result of the significant reduction of BAs in the intestine of STC patients, in turn forming a negative feedback, which inhibits intestinal absorption and increases the rate of liver synthesis; on the other hand, it also explained the significant inflammatory response and decreased motility in colon tissue of STC patients.

Host metabolism is affected not only by the microbial modification of BAs, which leads to changes in the signal pathways through the BA receptors, but also by the changing composition of the microbiota (Wahlstrom et al., 2016). Microbial deconjugation (removal of taurine or glycine conjugates) can prevent the reuptake of BAs through ASBT. BA deconjugation is carried out by Bacteroides with bile salt hydrolase activity (Ridlon et al., 2006). The enterotypes of Bacteroides are resistant to BAs, which grow in the presence of fat and bile (Arumugam et al., 2011; Tan et al., 2019). Studies have shown that Bacteroides coprophilus and Bacteroides thetaiotaomicron can act on the biotransformation of primary BAs, which are released in the intestine when dietary fat is ingested (Staley et al., 2017; Lynch et al., 2019). In our study, FXR in the colon tissues of STC patients was significantly reduced and a previous study also found that FXR deficiency enriched Desulfovibrionaceae (Sheng et al., 2017).

The pathological mechanism of STC is complicated. The amount of clinical samples currently included was not sufficient, and the analysis methods used also had certain limitations, which could not fully reveal the true metabolic state of the host. Future research should focus on targeted and precise quantitative analysis of relevant important differential metabolites and research on the cross-talk pathological mechanism between gut microbiota and the metabolites of the feces, so as to better guide clinical practice.

## Conclusion

In summary, the current analysis shows that STC patients exhibited imbalances in the intestinal flora. The α-diversity of the gut microbiota in the STC group was increased. β-diversity was markedly different from healthy subjects. The types of fecal metabolites that specifically changed in STC patients were determined. Further, the possible gut microbiota and metabolites with diagnostic value were screened out, and the interaction analysis was conducted. It may provide clues for a better understanding of STC’s intestinal microenvironmental change mechanism, and further experiments are needed to confirm their cross-talk. BAs and lipid metabolism seem to be an important link in the pathogenesis of STC and are, thus, worth further studying. This study provides an in-depth understanding of the relationship between the fecal microbiota, metabolites, and intestinal dysfunction in STC patients, and provides a possible future model for STC diagnosis and interventions targeting specific microbiota related to BA metabolism.

## Data availability statement

The datasets presented in this study can be found in online repositories. The names of the repository/repositories and accession number(s) can be found in the article/**supplementary material.**

## Ethics statement

This study was conducted in Tianjin Union Medical Center, Tianjin, China. The samples and clinical data used in this study were obtained with the approval of institutional review boards and under conditions of informed consent (China Clinical Trial Registry, ChiCTR2000033227; Medical ethics approval number: 2021B12). All subjects signed written informed consent upon enrollment and received questionnaires.

## Author contributions

YB and LY conceived and designed the project. YF, CX, LX, YW (13th Author), YL, HL, and LW collected samples. YF, CX, YW (4th Author), SZ, JA, and ZT did experiments and analysis. YF, LX, SZ, YL, YY, SY, BJ, and YW (4th Author) carried out data statistics. YF, CX, YL, YW (13th Author), and JA prepared figures. YF, CX, YB, and LY prepared and finished the manuscript. All authors contributed to the article and approved the submitted version.

## Funding

This work was supported by the National Key R and D Program of China (2018YFC1706506), the Foundation of Tianjin Municipal Health Commission (No. ZC20097), the Foundation of Tianjin Union Medical Center (No. 2020YJ017, 2017YJZD005), and the National Natural Science Foundation of China (No. 82174374).

## Acknowledgments

We greatly thank the patients and healthy volunteers who participated in sample collections.

## Conflict of interest

The authors declare that the research was conducted in the absence of any commercial or financial relationships that could be construed as a potential conflict of interest.

## Publisher’s note

All claims expressed in this article are solely those of the authors and do not necessarily represent those of their affiliated organizations, or those of the publisher, the editors and the reviewers. Any product that may be evaluated in this article, or claim that may be made by its manufacturer, is not guaranteed or endorsed by the publisher.
