# Supplementary figures and images for: Abnormal bile acid metabolism is an important feature of gut microbiota and fecal metabolites in patients with slow transit constipation
Source: Front Cell Infect Microbiol. 2022 Jul 28;12:956528. doi: 10.3389/fcimb.2022.956528 (PMC9366892; doi:10.3389/fcimb.2022.956528)

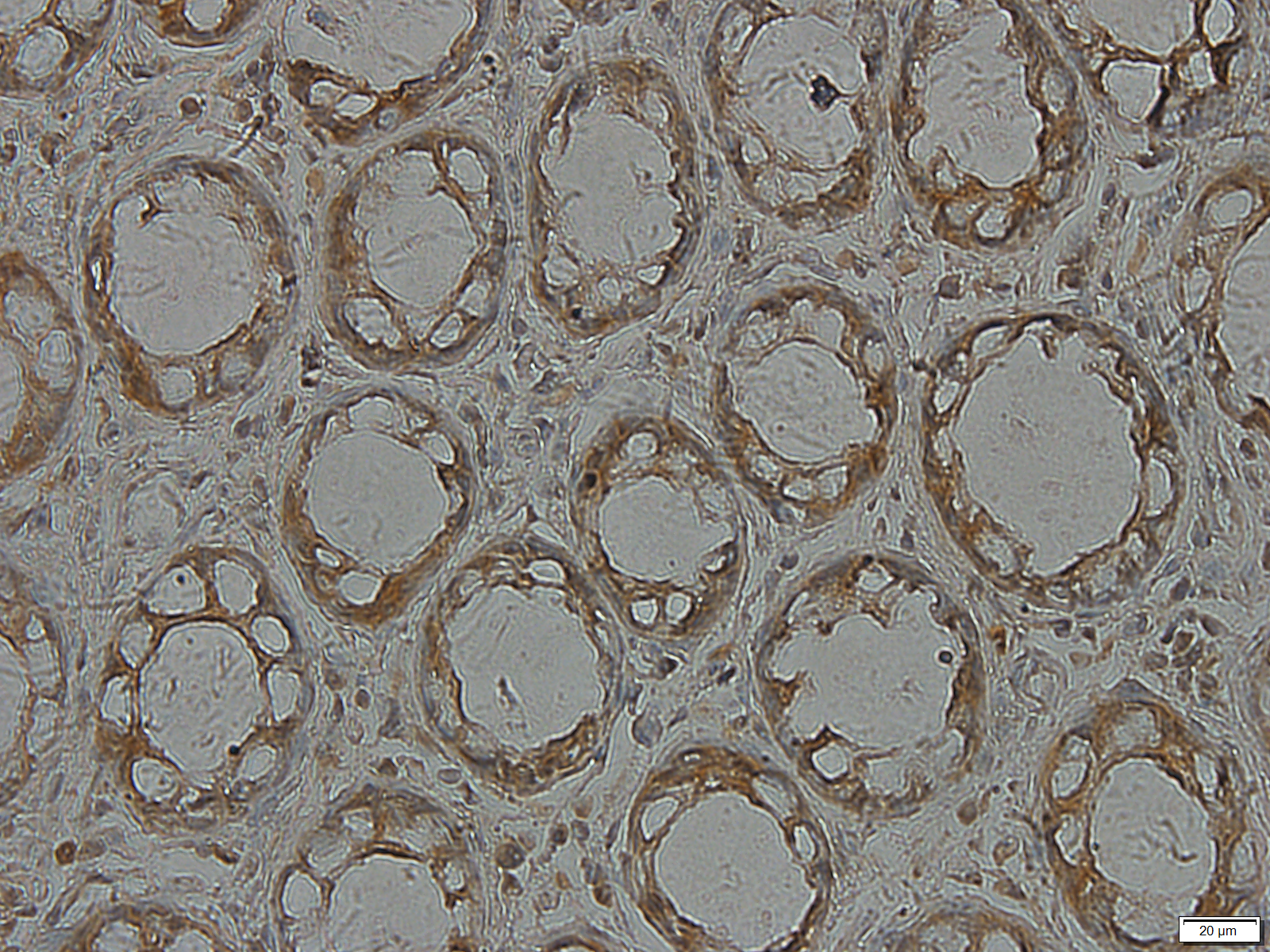

Supplement: Supplementary file 3 [file Image_2.tif]

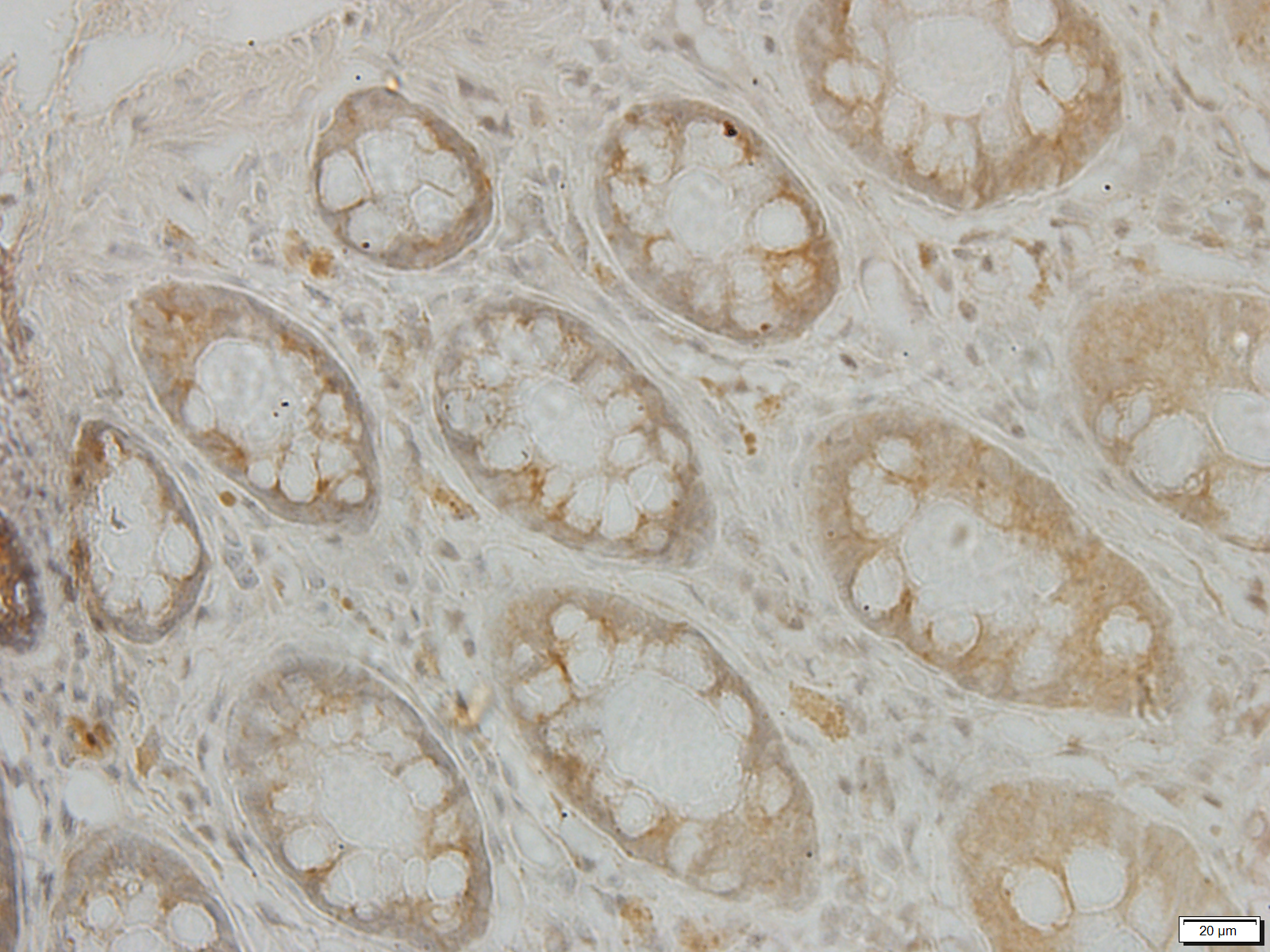

Supplement: Supplementary file 4 [file Image_3.tif]

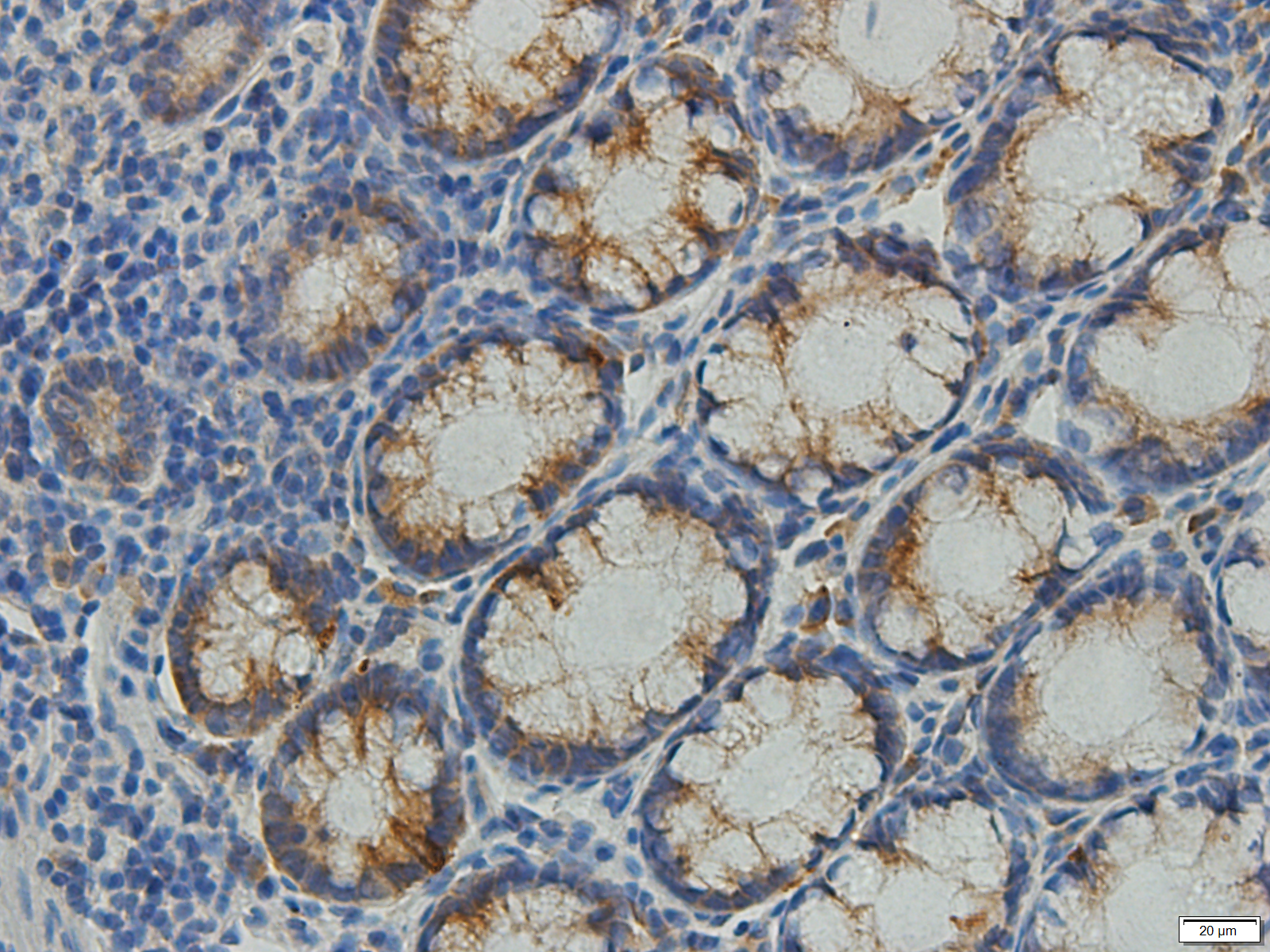

Supplement: Supplementary file 5 [file Image_4.tif]

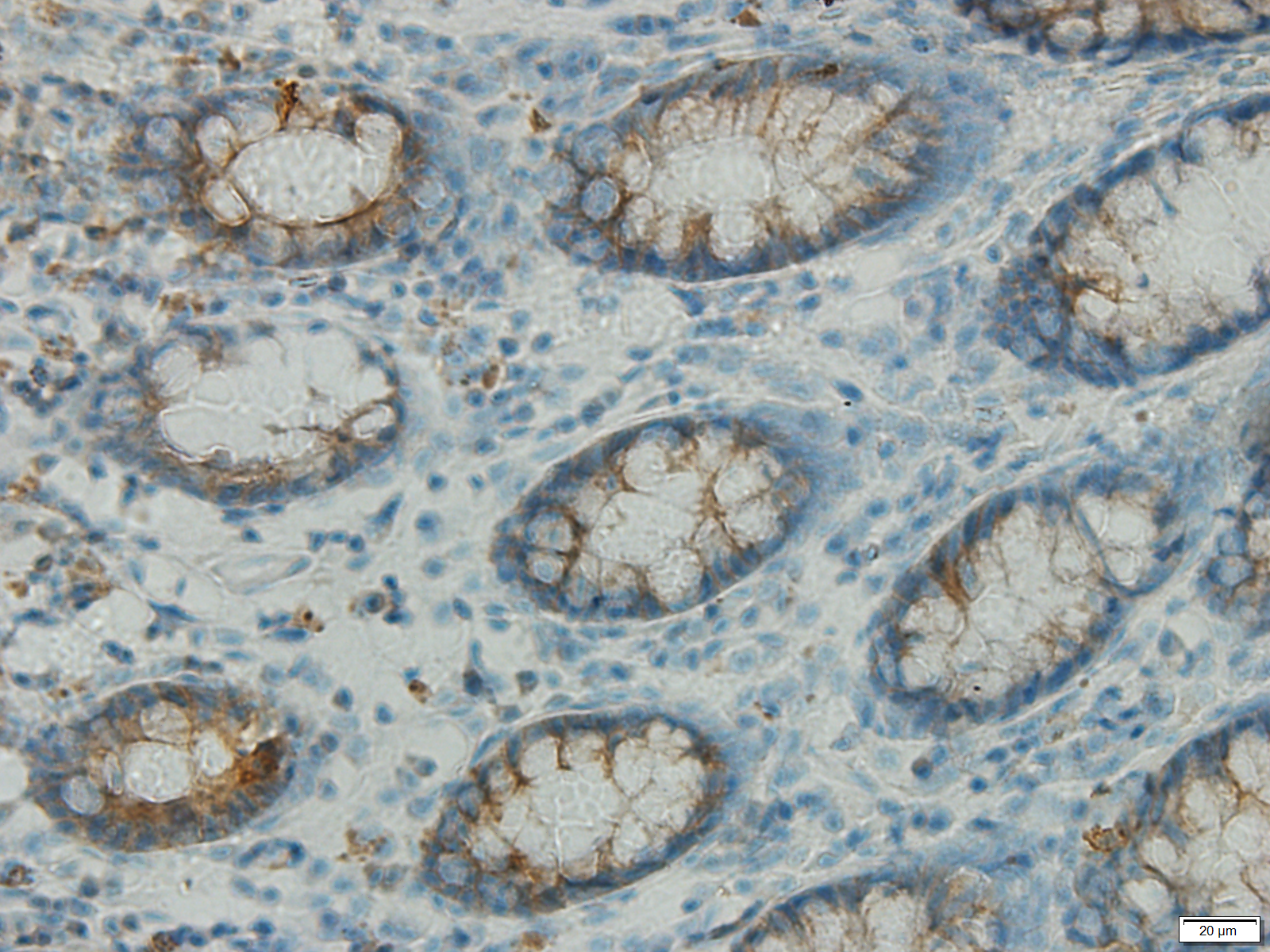

Supplement: Supplementary file 6 [file Image_5.tif]

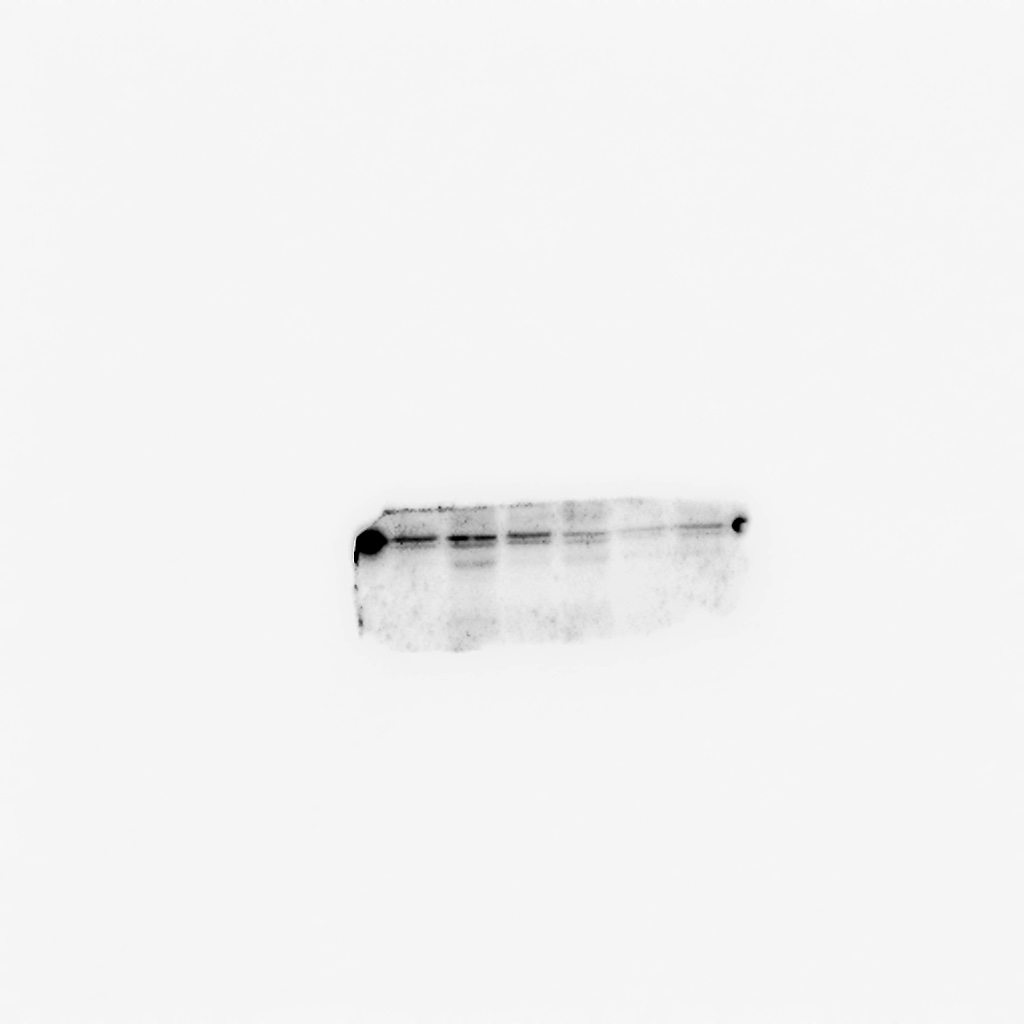

Supplement: Supplementary file 7 [file Image_6.jpeg]

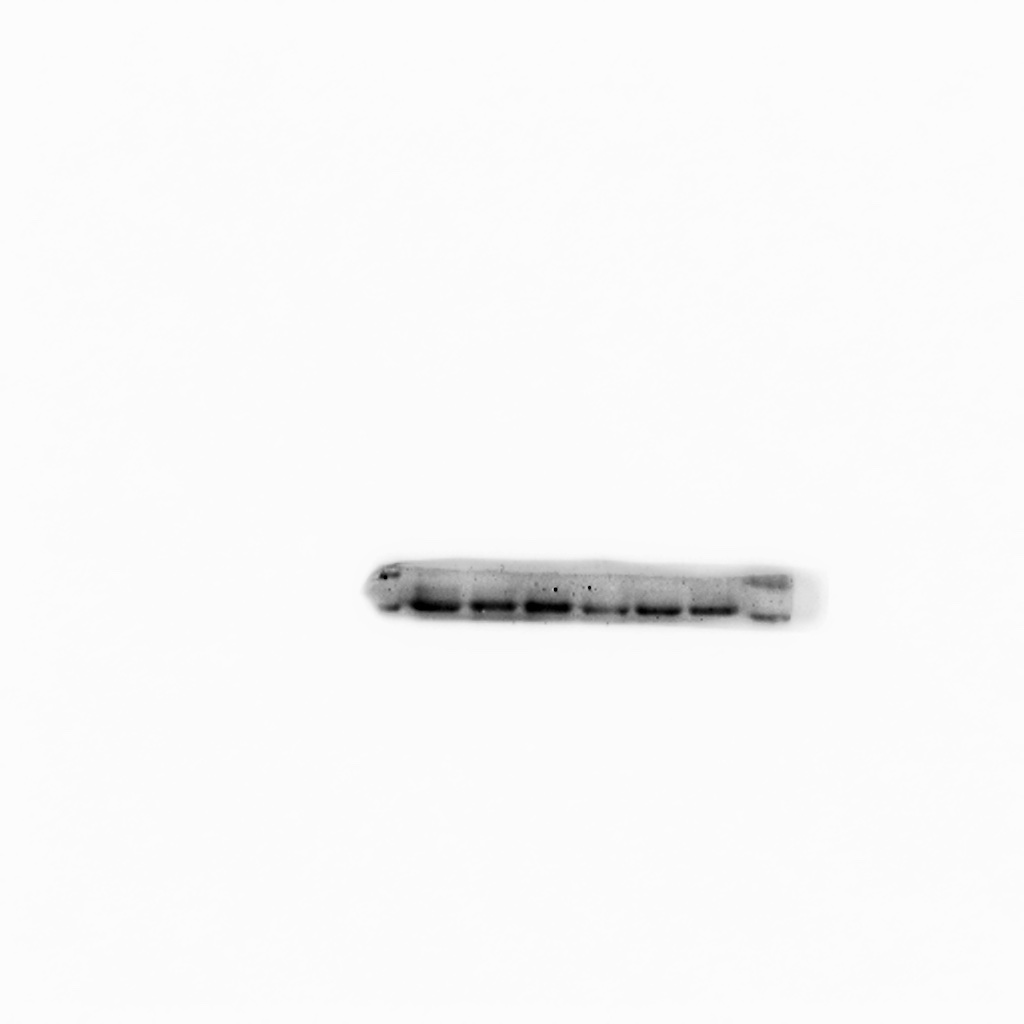

Supplement: Supplementary file 8 [file Image_7.jpeg]

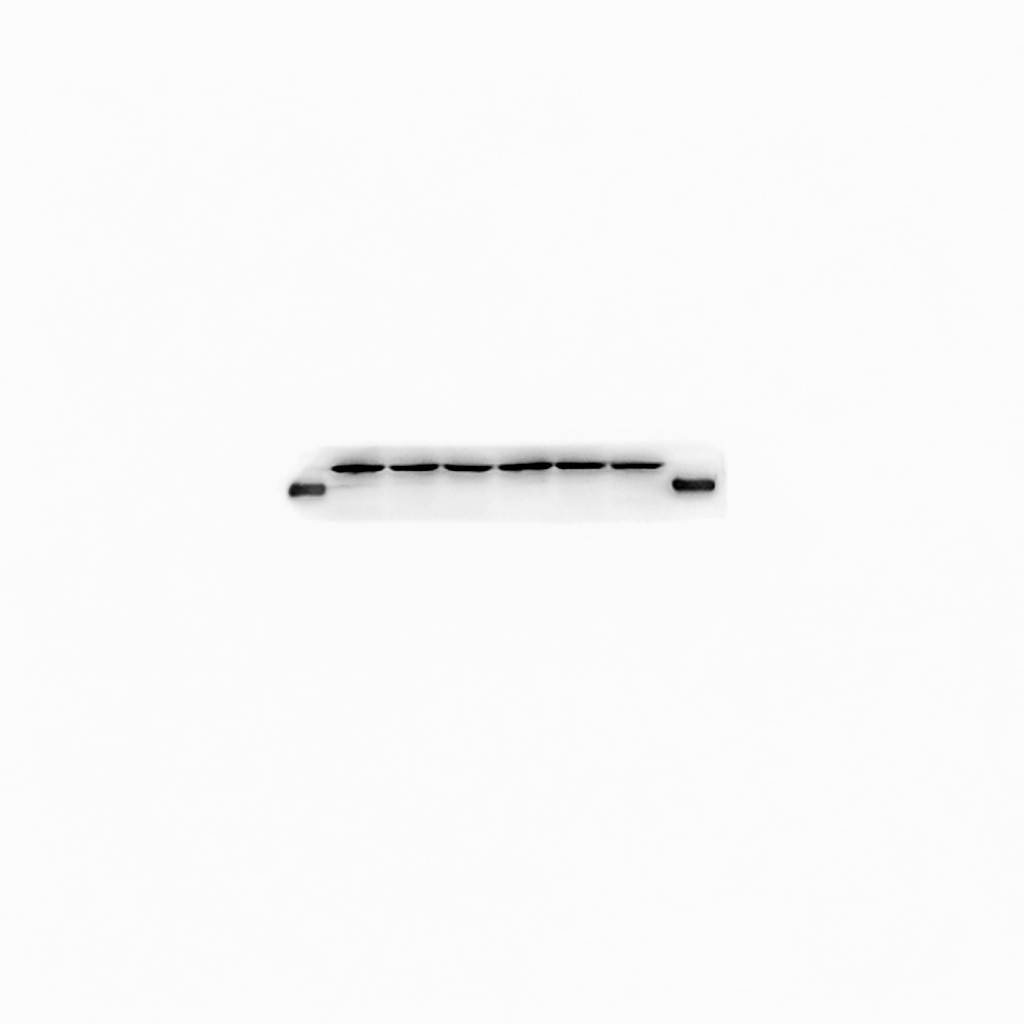

Supplement: Supplementary file 9 [file Image_8.jpeg]

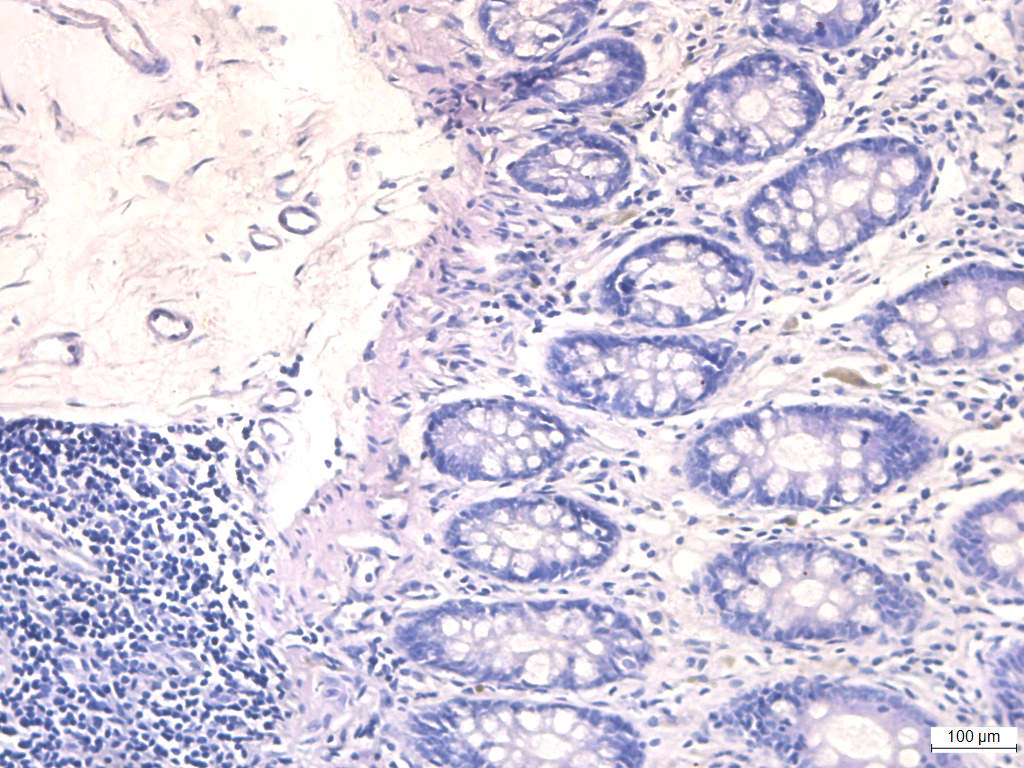

Supplement: Supplementary file 10 [file Image_9.tif]

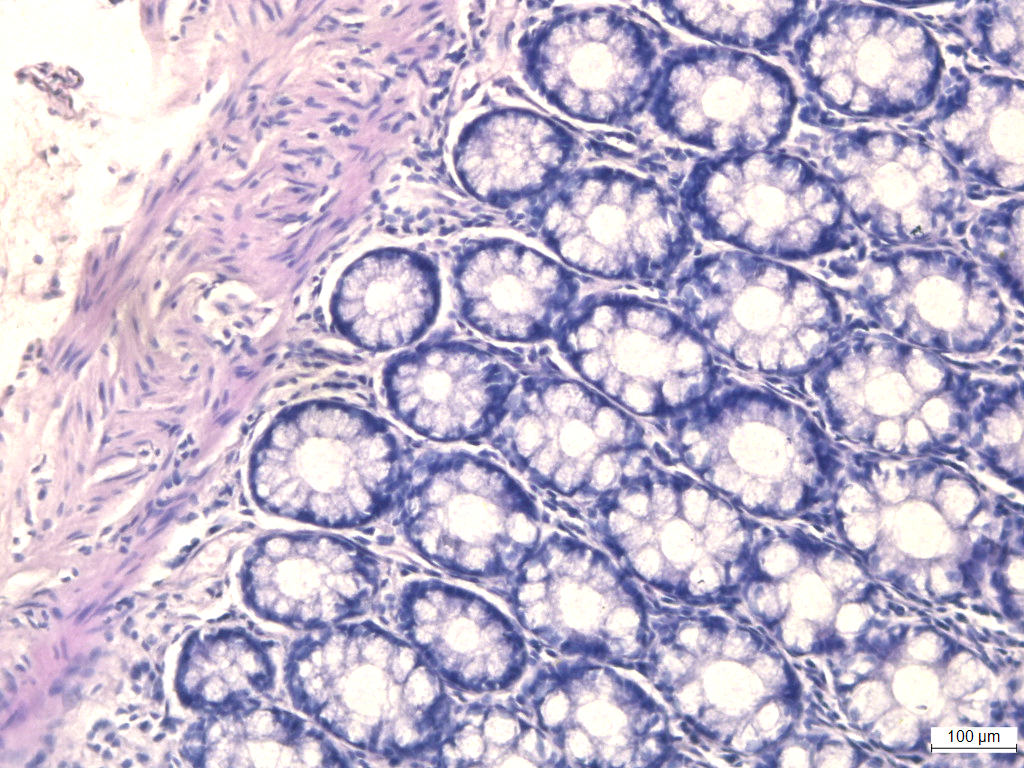

Supplement: Supplementary file 11 [file Image_10.tif]
